# Self-Regulated Learning and Academic Performance in Chilean University Students in Virtual Mode During the Pandemic: Effect of the 4Planning App

**DOI:** 10.3389/fpsyg.2022.890395

**Published:** 2022-05-26

**Authors:** Andrés Jaramillo, Juan Pablo Salinas-Cerda, Paula Fuentes

**Affiliations:** ^1^Escuela de Psicología, Facultad de Ciencias Sociales y Comunicaciones, Universidad Santo Tomás, Concepción, Chile; ^2^Escuela de Psicología, Facultad de Educación y Ciencias Sociales, Universidad Andrés Bello, Concepción, Chile; ^3^Escuela de Educación, Facultad de Educación, Universidad de Las Américas, Concepción, Chile

**Keywords:** self-regulated learning, mobile app, academic performance, college students, academic self-efficacy, 4Planning app

## Abstract

Research on the use of smartphone apps with the aim of developing *self-regulated learning* (SRL) and increasing academic performance of university students in virtual mode, as a result of the COVID-19 pandemic, is recent and scarce. The present article shows the results of a study that analyzed the effect of using the 4Planning app with an intra-curricular approach on SRL and on the academic performance of 119 1st-year psychology students in virtual mode, at a Chilean university (*M*_Age_ = 22.81, *SD*_Age_ = 6.52). The research was conducted with quantitative methodology and a quasi-experimental design, with pre- and posttest measurements of an experimental group and comparisons with three control groups. The results show that students who used the app during 10 weeks of the first academic semester 2021: (1) increased SRL self-report, (2) increased academic self-efficacy, and (3) obtained higher academic performance, compared to those who did not use it. The above occurs because the 4Planning app activates functions of the self-regulatory system of goal-directed behavior, which allows exercising the capacity of self-direction and self-influence on this type of behavior in the particular context of academic performance, which produces SRL. It is concluded that the 4Planning app is effective in developing self-regulation and improving the academic performance of 1st-year university students, in virtual learning mode.

## Introduction

The transition between secondary education and higher education is a critical stage, in which students may present shortcomings in competencies required for learning when they enter university, implying in many cases dropout or delay in educational trajectories (Dörrenbächer and Perels, 2016; Moreno-Marcos et al., 2020; Padilla-Petry and Vadeboncoeur, 2020; Sáez-Delgado et al., 2021). The development of competencies such as willingness to learn or autonomy requires personal, group, and institutional efforts (Sáez et al., 2018; Lobos et al., 2021a). In addition, the entry of the 2020 and 2021 generations of students occurred in particular conditions generated by the COVID-19 pandemic, due to the fact that countries adopted measures to deal with contagions and deaths (Organización Mundial de la Salud, 2020). Physical distancing, prevention measures, the vaccination process, and quarantine confinement have had a significant impact on the social, economic, physical, and mental aspects of people (Organización Mundial de la Salud, 2020; CEPAL, 2021). In the latter, increases in depressive, anxious, and academic stress symptoms have been identified, as well as alterations in sleep and eating routines, all of which affect the wellbeing of university students (Bao et al., 2020; Andrades-Tobar et al., 2021; Sekban and İmamoğlu, 2021).

The pandemic, the expansion of training processes in various areas and stages of life, and technological development and its daily access have driven the creation of virtual training environments. These environments offer innovative learning instances, because they imply changes in the teacher-student relationship, in the student–student relationship, in the access and management of information, in the strengthening of new competencies, etc. (Rivera-Vargas et al., 2021). Although people who begin to study in virtual environments tend to develop skills and competencies that allow them to adapt to these environments (Almusharraf and Khahro, 2020; Rivera-Vargas et al., 2021), teaching has not been standard for all, because it depends on technological barriers associated with socio-economic differences of students and their families, which exponentially increases the gaps in the conditions in which they learn (Almusharraf and Khahro, 2020; Tsolou et al., 2021). Institutions, teachers, and students worldwide have endured a rapid and forced change in the modality of teaching and learning generated by the pandemic, for which they were not prepared and which has had an impact on students’ experiences with their education (Lobos et al., 2021b). This new context has been called as *emergency remote teaching* (ERT) to differentiate it from other forms of teaching through virtual media, but which are planned and accessed voluntarily (Bozkurt and Sharma, 2020). Lobos et al. (2021b) investigated the beliefs of Chilean university students about their opportunities to learn and maintain their academic performance during the ERT period and found that most students presented negative beliefs about their learning (e.g., believing that their performance would be worse during this period).

Virtual learning environments intensified the role of the student as an active agent, because less external control is exercised over some aspects of their behavior (e.g., whether they are really paying attention to the class). Moreover, compared to secondary education, in higher education, there is also less external control, activities are more flexible, the relationship between teachers and students are more distant, and academic demands are more complex (Dresel et al., 2015; Sáez-Delgado et al., 2021). Therefore, the development of the capacity for self-regulation, the strengthening of autonomy, and the competencies and skills required for learning face-to-face, virtual, or hybrid modalities becomes more relevant (Almusharraf and Khahro, 2020).

However, in most generations entering university, a proportion of students present low levels of self-regulation of their learning process (Vassallo, 2011; Dresel et al., 2015). This ability is especially challenging to achieve medium- and long-term goals, as occurs in the academic context (Zimmerman, 2002), predicts the performance of university students (Alotaibi et al., 2017; Ergen and Kanadli, 2017), and is associated with dropout from the educational system of university (Bernardo et al., 2019; Bäulke et al., 2021) and high school students (Sáez-Delgado et al., 2021). *Self-regulated learning* (SRL) is the process by which people transform their mental capacities into academic skills, learning by themselves proactively, self-generating thoughts, feelings, and behaviors aimed at achieving the academic goals they set for themselves (Zimmerman, 2002; Panadero, 2017). The psychological processes involved in self-regulation are cognitive in nature. One example is forethought, which allow people to form beliefs about what they can do (*self-efficacy*), set goals, anticipate consequences of their eventual future actions, plan courses of action that produce the desired results, self-motivate, and guide their actions in an anticipatory and self-corrective manner.

Being a 1st-year student in higher education is a condition of potential risk, because novices, unlike those with greater expertise in an area, try to regulate their learning in a more reactive way, find it more difficult to set specific goals, monitor themselves systematically, and tend to attribute their performance to deficiencies in their skills, which is associated with lower personal satisfaction and defensive reactions. In addition, a smaller proportion of people who are initiated into a discipline immediately derive self-motivational benefits from SRL. In contrast, students with greater self-regulation vary their study methods and practices to discover new strategies for self-improvement, detect small gains in their learning, and increase their self-satisfaction and self-efficacy for performing at a high level of ability, so they increase the amount of study and practice, which predicts expertise (Zimmerman, 2002). In addition, those with greater expertise in an area tend to perform more SRL practices, compared to the more novice (Cleary and Zimmerman, 2001) and those with better performance use more of the sub-processes considered in Zimmerman’s model (DiBenedetto and Zimmerman, 2010).

Given that smartphone, tablet, or computer apps are easily accessible and everyday tools, especially by students (Kortabitarte et al., 2018; Hartley et al., 2020), studies have identified that using smartphones in the service of learning generates positive academic outcomes (Anshari et al., 2017; Nguyen et al., 2018; Ariel and Elishar-Malka, 2019; Dalvi-Esfahani et al., 2020) and that using them for non-academic purposes (e.g., to check virtual social networks) burdens students’ cognitive resource management by being a source of distraction when studying and when performing academic tasks outside of class (May and Elder, 2018; Aharony and Zion, 2019), which is associated with lower academic performance (Lepp et al., 2015; Chen and Yan, 2016). A small, recent body of research evaluates the impact of asking students to self-regulate their learning using apps (Xu et al., 2018; Foerst et al., 2019; Hendikawati et al., 2019; Kim et al., 2019; Loeffler et al., 2019; Broadbent et al., 2020; Hartley et al., 2020). Lobos et al. (2021a) used the self-regulation of learning model proposed by Zimmerman (2002) to design the 4Planning app for smartphone, with the aim of evaluating its impact on the SRL of 473 1st-year engineering students in face-to-face mode, belonging to seven Chilean universities, during the first semester of 2019. Using this app involves planning, evaluating, and correcting one’s own actions to achieve academic goals, in the context of a semester course, in intra-curricular modality. Lobos et al. used a quasi-experimental design, with pre- and posttest measurements of a control group and an experimental group, and identified that using the 4Planning app generates that students self-report a higher frequency of SRL practices, compared to those who do not use it.

As part of their research, Lobos et al. (2021a) systematically reviewed interventions that promoted SRL in college students using smartphones. While the seven studies they identified consider academic performance in argumentation, only two incorporated it as a dependent variable in their models (Xu et al., 2018; Hartley et al., 2020), but the proposed interventions did not generate a statistically significant impact. In addition, there is only one study of the effect of the 4Planning app on SRL, in a sample of engineering students, in face-to-face modality, without pandemic, and also did not consider performance as a dependent variable (Lobos et al., 2021a). According to Bandura (1977) and Zimmerman (2000), self-efficacy is sensitive to variations in the context in which students perform, which justifies investigating SRL in the particular modality of teaching and learning virtually, due to the COVID-19 pandemic. It is also unknown whether the frequency of use of the 4Planning app is linearly associated with academic performance. The ability to self-regulate is not a trait that is either possessed or not (Zimmerman, 2002), which requires measuring its presence as a continuous variable. Lobos et al. (2021a) did so categorically (uses vs. does not use the app). Also, longitudinal research is needed on the development of SRL skills, during more specific and shorter periods such as, for example, the 1st year of college (Panadero, 2017). Finally, research on these variables in the Chilean population is proportionally less than that generated in other countries. In this context, the aim of the present research was to analyze the effect of using the 4Planning app on the self-report of performing SRL practices, self-efficacy, and academic performance of 1st-year psychology students, from a Chilean university, in virtual mode due to the pandemic.

The 4Planning app was designed using the phased SRL model by Zimmerman (2002) as a framework, which is derived from Bandura’s *social cognitive theory of self-regulation* (Panadero, 2017). Bandura (1987, 1991) posits that the very cause of people setting goals and taking actions to achieve them lies in the fact that they possess a self-regulatory system, which gives them the capacity for self-direction and self-influence over their purposive behaviors. In order to achieve a goal, the intention and desire to reach it is not enough. It is necessary to exert self-influence on one’s own motivation and behavior, continuously reflecting on one’s own behavior and reacting to its consequences. Thus, people self-regulate their thoughts, feelings, motivations, and actions in the different contexts in which they set out to achieve goals.

Self-regulation of purposeful behavior occurs through four functions (Bandura, 1991). The *self-monitoring function* consists of paying adequate attention to one’s own performance, the conditions under which it occurs, and the immediate and distal effects it produces. It provides information for setting realistic goals and evaluating progress toward them. It also allows for self-diagnostics by identifying the significant characteristics of the social environment that explain our behavior. In addition, self-monitoring generates self-motivation, because when people pay continuous attention to how they perform, they set goals for progressive improvement, which generates evaluative self-reactions that mobilize efforts to achieve them. The second is the *judgmental function* and corresponds to the evaluation of one’s own performance using personally constructed standards, one’s own previous performance and/or that of other people in similar situations.

The third function of the self-regulatory system corresponds to *self-reactive influences*, through which we create incentives for our own actions and anticipate the affective reactions that will arise when we evaluate our performance. This has a regulating effect by affecting motivation, because when people condition their self-satisfaction on obtaining certain achievements, they motivate themselves to expend the necessary effort to obtain the desired performance. Thus, both anticipated satisfaction with desired achievements and dissatisfaction with insufficient achievements encourage actions that increase the probability of obtaining the expected performance. *Self-efficacy* is the fourth function of the self-regulatory system. People develop beliefs about their abilities to control their own functioning and the events that affect their lives. These beliefs influence their decisions, aspirations, effort, and perseverance in facing obstacles, whether their thought patterns are self-enhancing or self-enhancing, and their vulnerability to stress and depression. Thus, for example, the more self-efficacious a person considers him or herself to be, the higher his or her goals will be and the more committed he or she will feel to achieving them. On the other hand, those who doubt their capabilities are easily deterred by obstacles and failures.

In the context of academic performance, the activation of the functions of the self-regulatory system provides the capacity to exert self-direction and self-influence on behavior aimed at achieving academic goals, which generates SRL. For Zimmerman (2002), this type of learning occurs in three cyclical phases that affect each other. The *forethought phase* encompasses the processes that occur before efforts are made to learn. It includes task analysis, setting academic goals, and planning strategies to achieve them, as well as self-motivational beliefs, including self-efficacy, outcome expectations, intrinsic valuation given to a task, and to the learning process itself. The *performance phase* encompasses the processes that occur during the performance of learning efforts and includes self-monitoring and self-observation. Self-monitoring is exercised through imagery, self-instruction, focusing attention, and developing strategies to approach tasks. Self-observation, on the other hand, includes self-registration of personal events or those generated by self-experimentation, in order to identify the causes of such events and, thus, to act correctively if necessary. Finally, the *self-reflection phase* encompasses processes that occur after making efforts to learn and based on their results. In this case, there is the student’s self-judgment process, through self-evaluation and causal attribution of his performance. Also, there are the student’s self-reactions, in terms of self-satisfaction and positive affect, defensive responses to poor performance, or adaptive responses, such as making adjustments in the way of studying.

For Zimmerman (Panadero, 2017), the self-regulatory system and its functioning in the academic context as SRL correspond to a feedback loop. For cybernetic theory (García and Wittezaele, 1994), a system maintains its movement toward a goal, thanks to the fact that it possesses a flow of information about the effects of its actions and the degree to which it reaches the proposed goal. The actions and effects that bring it closer to the goal are maintained, while those that move it away are eliminated. Therefore, SRL implies producing and receiving information that allows one to exercise corrective self-control over one’s own actions, in order to achieve academic purposes.

As described in detail in the methodology of this study, learning to use the 4Planning app requires students to participate in nine group sessions, focused on learning outcomes that generate the activation of the functions proposed by Bandura (1991), as well as those considered in the SRL model proposed by Zimmerman (2002). From all of the above, it is expected that using the 4Planning app will have a positive effect on the self-report of SRL, self-efficacy, and academic performance, because it demands that students use and develop integrally their self-direction and self-influence capacities, planning their activities, exercising corrective, conscious, and deliberate self-control over their own cognitions, emotions, and actions related to their academic performance, which will allow them to perform the necessary activities to achieve the performance goals that they continuously and progressively set for themselves.

Consistent with the above, the present research hypothesizes that, between the pre- and posttest measurements, students who use the 4Planning app (experimental group), compared to those who do not use it (control group), will increase their self-report of SRL (*hypothesis 1*) and self-efficacy (*hypothesis 2*). Therefore, in the posttest, they will present higher self-report of SRL (*hypothesis 3*), higher self-efficacy (*hypothesis 4*), and higher academic performance (*hypothesis 5*). Furthermore, in the experimental group, the frequency of use of the 4Planning app will present a directly proportional relationship with the self-report of SRL (*hypothesis 6*), with self-efficacy (*hypothesis 7*), and with academic performance (*hypothesis 8*). Finally, SRL self-report and self-efficacy will present a directly proportional relationship with each other (*hypothesis 9*) and with performance (*hypotheses 10* and *11*, respectively).

## Materials and Methods

### Design and Participants

A quasi-experimental, longitudinal design was used. Participants were 119 Chilean 1st-year psychology students, in virtual teaching mode, from a private university located in the Biobio Region (*M*_Edad_ = 22.81, *SD*_Edad_ = 6.52). Students voluntarily self-assigned to the experimental condition (using the 4Planning app) or to the control condition (not using the app). For ethical considerations, participation was voluntary, so random assignment to the study conditions was not possible. The composition of both groups changed between the pretest measurement (March 2021) and the posttest measurement (July 2021). In the pretest, students in the experimental group (*n* = 48) and those in the control group (*n* = 49) who responded to the full battery of instruments, which included the SRL self-report and the academic self-efficacy scale, were considered. In the posttest, 50 students used the 4Planning app, but only 38 provided the SRL and self-efficacy self-report, while 30 students from a control group provided information on these same variables. Finally, the comparison of academic performance was made between the 50 students in the experimental group and 69 students in a control group.

### Instruments

#### Self-Regulated Learning Practices Scale

The SRL practices scale of Bruna et al. (2017) was used, which was constructed to describe the frequency of use of SRL strategies, corresponding mainly to the study readiness phase. It is composed of 11 items (e.g., “I identify what my study purposes are”) and the response format is Likert-type with seven response options (1 = never to 7 = always). Vergara et al. (2019) analyzed its psychometric properties in a population of university students and identified a one-factor model with an adequate fit (*N* = 716; CFI = 0.98; TLI = 0.97; RMSEA = 0.05). For their part, Lobos et al. (2021a) identified a reliability of 0.89 in the pretest application of their study. In the present investigation, the internal consistency (Cronbach’s alpha) in the pretest was 0.89 and in the posttest 0.90.

#### Academic Situations Specific Perceived Self-Efficacy Scale (EAPESA)

It is a unifactorial scale designed by Palenzuela (1983) to measure the global appreciation of competence in the academic environment. It is composed of 10 items (e.g., “I consider myself sufficiently capable to successfully face any academic task”) with a Likert-type response format with four alternatives (1 = never to 4 = always). Escobar and Pérez (2017) analyzed the psychometric properties of this scale in a sample of Chilean university students and identified a unifactorial structure and adequate internal consistency (Cronbach’s alpha = 0.87). In the present study, the reliability was 0.87 in the pretest and 0.88 in the posttest.

#### Academic Performance

It was measured with the evaluation instruments that each teacher designed to evaluate the contents of her subject. The evaluation scale ranges from 1 to 7 points, where the higher the score, the higher the academic performance.

#### Frequency of Use of the 4Planning App

The pedagogical sequence of the 4Planning app proposes the systematic progress of the student through a series of sessions with activities that must be developed stage by stage, as they achieve the objectives that each session establishes. Each student will advance according to his or her pace, motivation, and prioritization of work in the pedagogical sequence. This implies that not all students obtain the same level of progress or fulfillment of stages, which will be reported to the teacher at the time of feedback in the interface. We will call this progress compliance “frequency of use of the app.” The information is obtained through a report in an Excel table that indicates the level of progress of each student.

### Procedure

Within the framework of a teaching innovation project financed by the Universidad Andrés Bello, Chile, the use of the 4planning app was implemented in two 1st-year courses, in the day and evening programs of the psychology degree. Four teachers participated in four courses with a total of 200 students enrolled in the programs. The teachers worked together with the students on the pedagogical sequence of the app at the beginning of each class and during the week feedback was given through the application interface.

#### Description of the 4Planning App

The 4Planning app (Lobos et al., 2021a) was developed in the context of the project entitled “Intracurricular model for facilitating self-regulated learning competencies in university students,” which aims to promote the development of SRL strategies in university students. The app is particularly innovative because it involves the use of smartphones, making it intuitive to use. It presents a design focused on the user (young people), their needs and preferences. The pedagogical sequence includes didactic strategies of: (a) gamification, by obtaining scores, badges, and recognition messages, (b) transmedia narrative, which is deployed through multiple media, and (c) communicative tone adapted to the user, in the case of university students. Students participate in nine sessions, each associated with a specific learning outcome (see Table 1), in which they are invited to use different self-regulation strategies.

**Table 1:** 4Planning sessions and learning outcomes.

N	Session name	Learning outcomes
1	Study purposes	Reflects on his/her purposes of study (what he/she is.
2	Goals	Defines two goals for the subject, with respect to the purposes indicated in session 1.
3	Daily schedule for the week	Evaluates the distribution of time and makes
4	To-do list for the subject	Makes a list of things to do in the subject.
5	Development and prioritization of academic tasks	Updates daily to-do list.
6	Organization and balance of activities	Develops a to-do list according to importance and urgency.
7	Planning and preparing me individual study for assessments	Prioritizes to-do list items according to importance and urgency.
8	I plan and prepare my group study	Plan and prepare the group study.
9	I take advantage of learning in class	I fulfill basic behaviors for learning in class.
	Digital closure	I evaluate what I have learned.

Source: Lobos et al. (2021a).

The 4Planning app is used in intra-curricular mode, so that the sessions are developed in the context of one of the semester courses, through intentional actions of the teacher in the virtual classroom. In addition, the student, in an autonomous manner, works each session in his or her available time outside of class, thus strengthening the learning achieved in the classroom. The application consists of two environments, one for student management and the other for teacher management. The latter is in charge of reporting the students’ performance progress and is also the space where feedback on the actions performed by the students can be given. The feedback provided by the teacher to the inputs, comments, and/or activities submitted by the students through the app accompanies and guides the student’s process, allows instructions to be given, clarifies doubts, praises, and motivates them to continue completing the activities. Peers also provide feedback to each other.

Each session includes a motivational video, an infographic on the topic of the session, interaction activities with the app associated with the specific strategy of self-regulation of learning and an activity commitment that the student develops outside the virtual environment. The last session incorporates an activity where the student evaluates the usefulness of the experience of the developed session. In addition, the app delivers feedback messages that indicate to the student what to improve for future performance in each of the strategies promoted in each session.

### Data Analysis Strategy

To describe the study variables, measures of central tendency and variability were calculated (Keppel, 1991). To describe the variation of the study variables between the pre- and posttest, the Wilcoxon test for related samples was used. To compare the levels of the variables between the control group and the experimental group, the Mann–Whitney U test was used and to describe their relationship, Spearman’s correlation coefficient was used (Siegel and Castellan, 1972).

## Results

The Shapiro–Wilk test indicated that the SRL presented a non-normal distribution in the pretest in the experimental group (*p* < 0.01), in the posttest in this same group (*p* = 0.00), and in the control group (*p* < 0.05). The same was observed in the case of academic performance in the control group (*p* < 0.05). Therefore, abnormality was assumed for all study variables and nonparametric tests were used to contrast the hypotheses.

The descriptive data of the study variables are presented in Table 2. In the pretest, the Mann–Whitney U test for independent samples did not identify statistically significant differences between the control group and the experimental group in the levels of SRL (*p* > 0.05) or self-efficacy (*p* > 0.05). In the posttest, the experimental group presented higher SRL (*p* < 0.05), higher self-efficacy (*p* < 0.05), and higher performance (*p* < 0.01), compared to the control group. In addition, the Wilcoxon test for related samples indicated that, between the pre- and posttest, the experimental group increased SRL (*p* < 0.01) and self-efficacy (*p* < 0.01), while the control group did not increase SRL (*p* > 0.05) or self-efficacy (*p* > 0.05; Table 2).

**Table 2:** Descriptive data of the study variables in the control group and in the experimental group.

Variable	Control group				Experimental group			
	*n*	*M*	*Md*	*SD*	*n*	*M*	*Md*	*SD*
**Pre-test**								
Self-report of self-regulated learning	49	5,03	5	1,09	48	5,12	5,27	1,18
Academic self-efficacy	49	1,85	1,8	0,48	48	1,84	1,9	0,46
**Post-test**								
Self-report of self-regulated learning	30	5,05	5,45	1,25	38	5,71	6,09	1,03
Academic self-efficacy	30	1,86	1,9	0,48	38	2,15	2,1	0,43
Frequency of use of 4Planning	-	-	-	-	50	7,8	5	5,95
Academic achievement	69	5,15	5,5	1,15	50	5,87	6,05	0,99

Table 3 shows the Spearman correlation coefficients between the study variables identified in the posttest in the experimental group. The frequency of use of the 4Planning app presented a positive and medium correlation with academic performance (*r* = 0.38**, *p* < 0.01; *n* = 50), a small, positive correlation with self-reported SRL (*r* = 0.28*, *p* < 0.05; *n* = 38), and a non-significant correlation with academic self-efficacy (*r* = −0.13, *p* > 0.05; *n* = 38). The SRL showed a positive, small, and non-significant correlation with performance (*r* = 0.24, *p* > 0.05; *n* = 38) and with self-efficacy (*r* = 0.22, *p* > 0.05; *n* = 38). The latter variable showed a positive correlation of medium size with performance (*r* = 0.29*, *p* < 0.05; *n* = 38).

**Table 3:** Spearman’s correlation coefficient between the study variables in the post-test in the experimental group.

Variable	1	2	3	4
1. Frequency of use of 4Planning	-			
2. Self-report of self-regulated learning	0.28^*^	-		
3. Academic self-efficacy	−0.13	0.22	-	
4. Academic achievement	0.38^**^	0.24	0.29^*^	-

* Significant at the 0.05 level.

** Significant at the 0.0 level.

## Discussion

Research on the effect of smartphone apps on the self-regulation of learning and academic performance of online students in the pandemic mode is scarce and recent. In this context, the main results of this study indicate that 1st-year psychology students who used the 4Planning app to develop their SRL, during 10 weeks of the first academic semester of 2021, in virtual mode as a result of the pandemic, obtained higher performance academic performance, higher self-report of performing SRL practices, and higher academic self-efficacy, compared to students who did not use it. These results are discussed below.

Using the 4Planning app generated higher academic performance compared to not using it, which supports hypothesis 5, and the higher the frequency of app use, the higher the academic performance, in accordance with hypothesis 8. The size of this effect is medium and is detected despite the fact that both variables presented dispersions of different magnitude, which makes it difficult to model their relationship linearly. Unlike previous studies that have attempted to improve learning by using apps (Xu et al., 2018; Hartley et al., 2020), both results of the present study show that the 4Planning app generates a qualitative and quantitative change in the performance of those who use it. Similar to how Woottipong (2022) explains the results of his intervention, aimed at developing the SRL of university students in a context of face-to-face and virtual interaction, the effect of the 4Planning app on academic performance occurs because it activates in an integrated manner different functions and processes involved in the self-regulation of learning. Thus, for example, reflecting on the purposes of study (Session 1) requires the use of self-monitoring in the forethought phase. In this phase, students made an intrinsic assessment of the task to be addressed and of the learning process itself, analyzed the nature of the task, and activated self-efficacy beliefs, as well as expectations of results. All of the above contributed to the setting of more realistic and progressively demanding goals (Session 2). Both processes generated self-motivation and positive disposition to learning and the selection of actions aimed at achieving the goals set, which were distributed in specific weekly schedules (Session 3). These learning outcomes, as well as those of all app implementation sessions, are produced by a self-regulated learner (Zimmerman, 2002), because they involve as: (a) setting attainable goals, (b) intensively using strategies to achieve them, (c) self-monitoring performance looking for signs of progress, (d) restructuring the social and physical context to be compatible with goals, (d) effectively managing personal time, (f) self-evaluating the methods used, (g) attributing causes to the performance achieved, and (h) adapting future methods.

The presence of other factors also explains the dispersion of academic performance observed within the group of students who used the app. The students took the course from start to finish, so that during the semester they activated, to some degree, all the psychological functions considered in Zimmerman’s three-phase model, also affecting academic performance. However, the 4Planning app focuses on the preparatory thoughts phase, so that the psychological processes conceptualized in the other phases were not under experimental control during the app implementation sessions. It is suggested that future versions of this app propose learning outcomes for all three phases of the model, so that the stimulation of self-regulated learning better fits its circular nature (Zimmerman, 2002). In this way, it could be experimentally evaluated whether, taking into account the above, the app expands its influence on the SRL and academic performance of those who use it in a virtual and in a face-to-face learning context.

A second result indicates that, between the pre- and posttest, students who used the 4Planning app increased self-reported SRL compared to those who did not use it, which supports hypothesis 1. This result is similar to that identified by Lobos et al. (2021a) with Chilean engineering students and is consistent with the results of studies that have also used apps to develop SRL of Indonesian (Hendikawati et al., 2019) and Australian (Broadbent et al., 2020) university students. Furthermore, in accordance with hypothesis 3, in the posttest, the self-report of SRL was higher than that of students who did not use the app. Finally, the frequency of app use presented a directly proportional relationship, of small size, with this self-report, which supports hypothesis 6. Using the 4Planning app requires students to perform self-regulatory practices, while the SRL self-report scale indicates the degree to which students consider that they performed such practices. Therefore, the self-report of performing SRL practices increased because students indeed increased the frequency of self-regulatory practices, which was ascertained by them employing self-monitoring and self-observation. However, both functions are selective attention processes and the authors of the main SRL models consider that the psychological functions involved in self-regulation, can also operate automatically, implicitly, or non-consciously for the learner (Panadero, 2017), so that an absolute correspondence between the actions effectively performed and the self-reported ones are not to be expected.

The third result indicates that the 4Planning app increased the academic self-efficacy of the students who used it, compared with those who did not use it, which supports hypothesis 2. Although none of the app implementation sessions contemplated strengthening self-efficacy beliefs as a learning outcome, students may have considered that using the app would help them achieve their goals. In addition, using the 4Planning app engages students in exploration and awareness of their capabilities (i.e., self-monitoring), which nurtures their beliefs about their ability to achieve academic goals. By seeing themselves involved in setting goals and taking actions to achieve them, their self-efficacy beliefs would have developed positively. This is due to the fact that the set of psychological functions involved in self-regulation constitutes a system, so that the modification of one of its parts affects the others and the whole system, just as the functioning of the whole system affects its parts (García and Wittezaele, 1994). For this same reason, SRL self-report and academic self-efficacy presented a positive correlation with each other, which supports hypothesis 9 of the study and is consistent with previous studies that relate both variables (Zimmerman and Schunk, 2008; Woottipong, 2022). For example, Follmer (2022) designed an SRL strategy for use by undergraduate students in a statistics course and identified that those who used it obtained higher academic self-efficacy and greater learning of statistics. On the other hand, the literature shows that students tend to meet the performance expectations that teachers form of them (Gentrup et al., 2020). During the implementation sessions, the students received support and encouragement from the course teachers, who had a high level of conviction about their own ability to guide the students and the positive effect using the app would have, which contributed to developing their beliefs about their own ability to achieve the goals they set for themselves.

The frequency of use of the 4Planning app was not linearly associated with academic self-efficacy, which does not support hypothesis 7. Self-efficacy develops, is specific to performance domains, and depends on different factors (Bandura, 1977; Zimmerman, 2000), which allows us to hypothesize that, during the semester, students were elaborating their self-efficacy beliefs about the course in which the use of the app was incorporated. The cyclical nature of the self-regulation of learning produces that the self-reflection associated with previous efforts to learn affects subsequent forethought. Thus, for example, the self-reaction of dissatisfaction with one’s own performance leads to lower levels of self-efficacy and less effort to learn (Zimmerman, 2002). Regarding the above, Winne posits that “a vital feature of SRL is cycles of information flows rather than a uni-directional flow of information. Some cycles are internal to the person and others cross the boundary between person and environment” (Panadero, 2017, p. 21). For his part, Zimmerman (Panadero, 2017, p. 20) states that as:

My cyclical model of SRL elaborates these triadic components and describes their interaction in terms of repeated feedback cycles. Thus, any variable in this model (e.g., a student's self-efficacy beliefs) is subject to change during the next feedback cycle.

Self-efficacy beliefs determine how people explain their successes and failures. Those who consider themselves highly effective tend to attribute their failures to insufficient effort, while those who consider themselves ineffective attribute it to low ability. In addition, they influence decisions, aspirations, effort, and perseverance in facing obstacles, whether thinking patterns are self-enhancing or self-supportive and vulnerability to stress (Bandura, 1991). Therefore, the students’ academic performance during the semester, as well as their self-reactions, determined the other two phases, for better and for worse. Those who obtained less than expected could have decreased their self-efficacy and their efforts to learn, while those who obtained more could have increased them. All of the above show that (holding other factors constant) the cyclical nature of the self-regulation process, and the nature of self-efficacy, can contribute to or deteriorate academic performance (Panadero, 2017). Self-efficacy beliefs can distort the “objective” information available to achieve a goal. Unlike a material system (e.g., a freezer), people have an opinion about their ability to perform actions and achieve goals. Therefore, they may underestimate or overestimate this capacity, which explains the occurrence of phenomena such as positive synergy and negative synergy, in which it is observed that performance is not completely predictable from the resources that an observer can identify in a person.

Another result identified that SRL self-report presented a small correlation with academic achievement, which supports hypothesis 10. The scale used in the present study to measure SRL (Vergara et al., 2019), was developed in a hypothetico-deductive manner, considering the literature and expert judgment, and focuses on the first phase of Zimmerman’s model (Lobos et al., 2021a). However, the more processes of the different phases of this model are stimulated, the higher the performance (Cleary et al., 2006). Therefore, the students’ academic performance was also influenced by self-regulatory practices not considered in this scale, since it contemplates only a sample of the total number of possible practices. It is pertinent to continue inductively identifying other self-regulatory practices used by students with high learning levels and to evaluate their impact using an experimental design. Doing so would allow prescribing a greater diversity of self-regulatory practices that increase the prediction and theoretical understanding of academic performance.

The last result of the present study showed that self-efficacy and academic performance presented a positive correlation, which supports hypothesis 11. This result is consistent with that of reviews showing that both variables covary in different student populations (e.g., Multon et al., 1991; Honicke and Broadbent, 2016). Also, it coincides with those reported in Latin American (Alegre, 2014) and non-Latin American (Follmer, 2022) populations.

The results of the study show that the frequency of use of the 4Planning app presented a higher magnitude covariation with academic performance, compared to the correlation observed between SRL self-report, self-efficacy, and performance. Bandura (1991) states that motivation and self-efficacy are necessary, but not sufficient to achieve a goal. It is necessary to engage, effectively, in self-regulatory practices of the actions that make it possible to achieve it. The SRL scale provides the students’ self-report of the frequency with which they consider that they executed certain self-regulatory practices. On the other hand, the indicator of use of the 4Planning app is the record that the system provides of the frequency with which they actually carried out such practices. Therefore, the best predictor of academic performance was the actual and practical involvement in self-regulatory behaviors.

The results of the present investigation should be interpreted considering some limitations. The small sample size did not allow us to perform statistical analyses that have greater statistical power and require normally distributed variables. For example, multiple regression to identify the predictive capacity of our study variables with respect to academic performance. In addition, three control groups of students were considered because the participation changed during the semester and students were self-assigned to the study conditions and the sample selection was non-random. However, self-assignment did not generate that, in the pretest, the experimental group presented statistically significant differences in the self-report of SRL nor in self-efficacy. Furthermore, in the pretest, in the experimental group, the self-report of SRL and self-efficacy did not present significant associations with the use of 4 Planning (both *p* < 0.01). On the other hand, the effect of the 4Planning app on SRL identified in the present research was the same as that identified by Lobos et al. (2021a) using a control group that did not change its composition between the pretest and the posttest and with a large sample composed of 473 engineering students from seven Chilean universities, located in different geographical and sociocultural contexts.

In general terms, the results of this study, contextualized in the line of research where they are inserted, show that the joint work of teachers and students supported by the use of accessible technology and everyday use by current generations, develops self-regulation, and increases the academic performance of students from careers as dissimilar as engineering and psychology. The foregoing shows that the capacity for self-regulation is a transversal competence and that its development can take place in a relatively short time at the beginning of higher education, in face-to-face learning mode, and also in virtual mode.

In particular terms, the results of the present study constitute empirical evidence in favor of the ecological validity of the hypothesis of the effect of the 4Planning app on the SRL and on the academic performance of 1st-year higher education students in virtual mode. This effect occurs because using the app activates functions of the goal-directed behavior self-regulation system, which allows exercising the capacity for self-direction and self-influence in the particular context of academic performance. The app is versatile, communicative, and includes gamification. It is recommended for use in an intra-curricular context, for adolescents and young adults entering higher education, to develop their SRL and increase their academic performance. The results of the study confirm that to achieve a goal it is not enough to desire it, but it is necessary to act purposefully because “inspiration exists, but it must find you working” (Pablo Picasso, w/i).

## Data Availability Statement

The raw data supporting the conclusions of this article will be made available by the authors, without undue reservation.

## Ethics Statement

Ethical review and approval was not required for the study on human participants in accordance with the local legislation and institutional requirements. The participants provided their written informed consent to participate in this study.

## Author Contributions

AJ contributed to designing the study, reviewing the literature, designing and data analysis, interpreting the results, discuss the results, and write and revise the manuscript. JS contributed to designing the study, reviewing the literature, and write and revise the manuscript. PF contributed to the implementation of the experiment and the revision of the manuscript. All authors contributed to the article and approved the submitted version.

## Funding

This research was supported by the Research Directorate of the Universidad Andrés Bello and by the teaching innovation project of the Teaching Directorate of the Academic Vice-Rectory of the Universidad Andrés Bello.

## Conflict of Interest

The authors declare that the research was conducted in the absence of any commercial or financial relationships that could be construed as a potential conflict of interest.

## Publisher’s Note

All claims expressed in this article are solely those of the authors and do not necessarily represent those of their affiliated organizations, or those of the publisher, the editors and the reviewers. Any product that may be evaluated in this article, or claim that may be made by its manufacturer, is not guaranteed or endorsed by the publisher.
